# Molecular Mechanism of L-Pyroglutamic Acid Interaction with the Human Sour Receptor

**DOI:** 10.4014/jmb.2212.12007

**Published:** 2022-12-30

**Authors:** Sanung Eom, Shinhui Lee, Jiwon Lee, Minsu Pyeon, Hye Duck Yeom, Jung Hee Song, Eun Ji Choi, Moeun Lee, Junho H Lee, Ji Yoon Chang

**Affiliations:** 1 Department of Biotechnology, Chonnam National University, Gwangju 61186, Republic of Korea; 2 Research and Development Division, World Institute of Kimchi, Gwangju 61755, Republic of Korea

**Keywords:** L-Pyroglutamic acid, sour taste, hPKD2L1, Xenopus oocyte, two-electrode voltage clamp

## Abstract

Taste is classified into five types, each of which has evolved to play its respective role in mammalian survival. Sour taste is one of the important ways to judge whether food has gone bad, and the sour taste receptor (PKD2L1) is the gene behind it. Here, we investigated whether L-pyroglutamic acid interacts with sour taste receptors through electrophysiology and mutation experiments using *Xenopus* oocytes. R299 of hPKD2L1 was revealed to be involved in L-pyroglutamic acid binding in a concentration-dependent manner. As a result, it is possible to objectify the change in signal intensity according to the concentration of L-pyroglutamic acid, an active ingredient involved in the taste of kimchi, at the molecular level. Since the taste of other ingredients can also be measured with the method used in this experiment, it is expected that an objective database of taste can be created.

## Introduction

Taste is generally classified into five flavors: salty, sweet, bitter, umami, and sour [1]. The detection of sweetness is a survival strategy as it is necessary to determine which foods can be used as an energy source [2]. Bitter taste detection has evolved as a way to distinguish whether a food is poisonous [3]. In this way, the ability to taste flavors has evolved for the purpose of gaining information necessary for human survival. For example, sour taste evolved to enable us to distinguish whether food is rotten or not [4]. The ability to recognize and distinguish flavors is also an important factor in satisfying our appetite, which is indispensable for happiness. However, the mechanism behind how we determine taste has not been revealed.

Since Dr. Charles Zuker characterized his five taste receptors in the 2000s [5-7], research on taste receptors has gained traction. The genes involved in the five major tastes were discovered and announced as follows:.sour taste [4] binds to the PKD2L1 membrane protein, and signals are transmitted to TAS1R1/TAS1R3 for sweet taste [8], TAS1R2/TAS1R3 for umami taste [9], TAS2R16 for bitter taste [10, 11], and ENaC for salty taste [12]. Using these genes, researchers focused more deeply on each taste, and studies on taste receptors for representative foods are also being conducted. However, no study so far has analyzed the taste of the active ingredients in kimchi through the taste receptors.

Kimchi is a traditional Korean fermented food that has been recognized for its health benefits since ancient times. In addition, metabolites produced by fermentation through lactic acid bacteria are known to play a leading role in health [13]. In fact, various studies involving animal experiments have been published and showed that the active ingredients in kimchi have antioxidant activity [14], help in cognitive improvement [15], and exert anticancer effects [16-18]. However, it has not been revealed at the cellular and molecular level what kind of taste these ingredients of kimchi represent.

L-Pyroglutamic acid (LPGA) is one of the active ingredients of kimchi When *Thermophilic lactobacillus* is inoculated, more LPGA is produced [19]. LPGA is a metabolite of the glutathione cycle that is converted to glutamate by 5-oxoprolinase and is a natural amino acid derivative that has been rarely studied due to its high cost [20, 21]. It has a moisturizing function and is used as a moisturizer component [22, 23]. In addition, LPGA is known to have salty, umami, and sour flavors [24]. The molecular mechanism of the salty taste and the umami taste was confirmed by activating each taste receptor [25, 26]. However, it has not been found how LPGA acts on sour taste receptors.

In the present study, we investigated whether L-pyroglutamic acid binds to hPKD2L1, a human sour taste receptor, and produces a sour taste at the molecular level. As a result, LPGA interacted with hPKD2L1, and the concentration gradient characteristics and the binding site could be specified. Through these results, we revealed for the first time at the molecular level that LPGA has a sour taste, and the degree of signal intensity change according to the amount is shown as an objective value.

## Material and Methods

### Materials

**Fig. 1:**
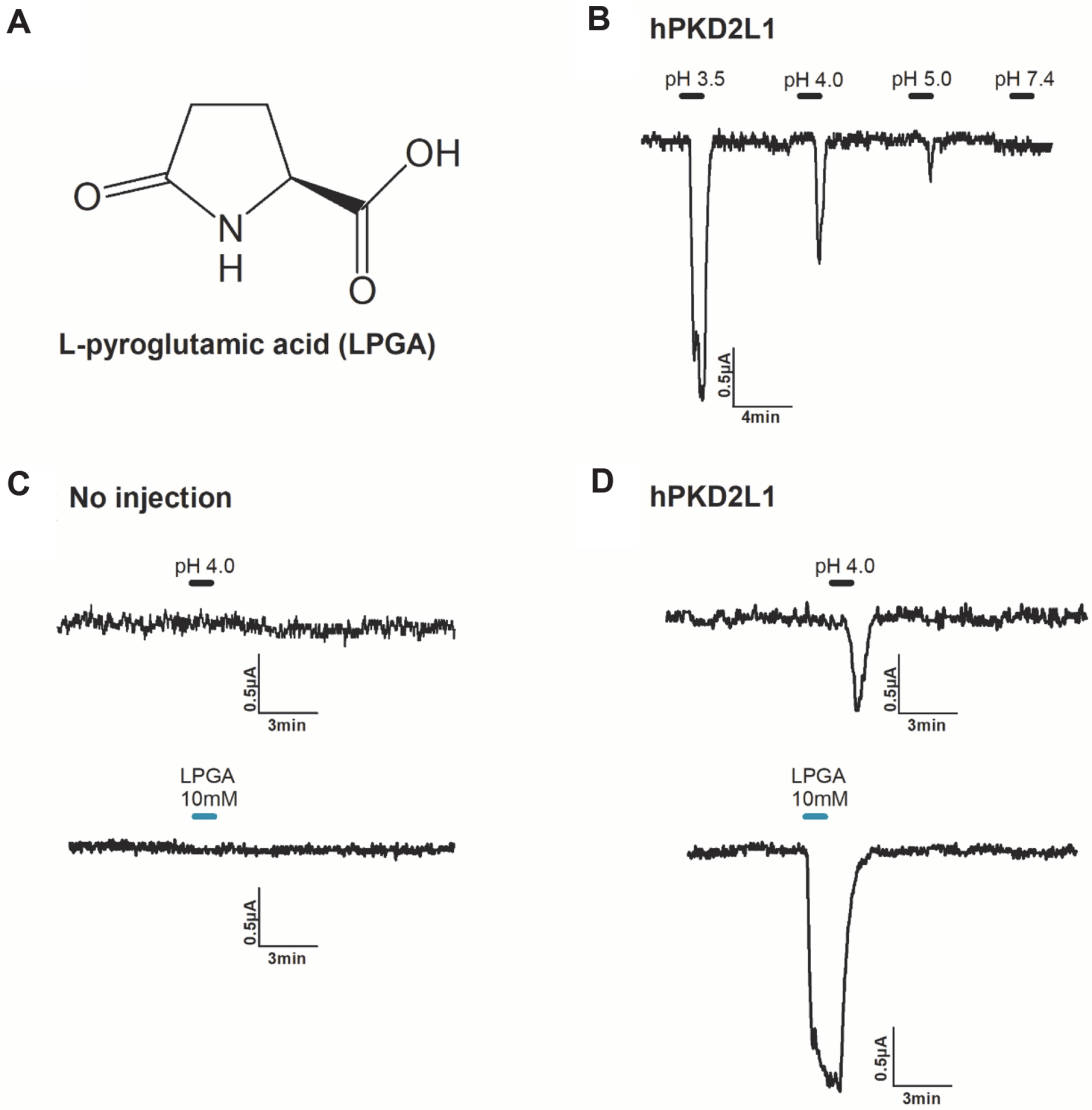
Confirmation of interaction between LPGA and hPKD2L1. (**A**) The chemical structure of LPGA. (**B**) Confirmation of proton concentration-dependent activity of hPKD2L1. (**C**) By applying proton and LPGA to non-injected oocyte, it was confirmed that no activity was shown in non-injected oocytes. (**D**) In oocytes injected with hPKD2L1 and expressed, activity by proton and LPGA is shown. The scaling bar and each applied pH are displayed at the top side of the current in the figures. All experiments were performed at room temperature, and the holding potential of the protocol used in the experiments was -80 mV. (*n* = 6-8, obtained from four different oocytes)

L-Pyroglutamic acid (Merck Korea, Korea) was dissolved in a dimethyl sulfoxide (DMSO) solvent and diluted to prepare a recording solution for the next experiment (less than 0.01% DMSO in the final recording solution). Fig. 1A shows the chemical structure of L-pyroglutamic acid. The human PKD2L1 cDNAs (GenBank Accession No. NM_016112) were purchased from OriGene (USA). All other compounds were supplied by Sigma (Merck Korea).

### *Xenopus* Oocyte Culture and Microinjection

Surgery was performed to manually collect oocytes of *Xenopus*, which were then separated into single cells by treatment with 0.5 μg/ml of collagenase within 2 h. Stage V or VI oocytes in good condition were selected from single oocytes using Ringer's solution (82.5 mM NaCl, 2 mM KCl, 1 mM MgCl2, 5 mM HEPES; pH 7.35). Afterward, the oocytes were stored in ND96 incubation solution (96 mM NaCl, 2 mM KCl, 1.8 mM CaCl2, 1 mM MgCl2, 5 mM HEPES, 2.5 mM sodium pyruvate, 50 mg/ml gentamicin solution; pH 7.35) at 15°C. According to the Chonnam National University Guidelines for Animal Care (CNUIACUC-YB-2016-07), *X. laevis* were cared for and handled in adherence to the Korean *Xenopus* Resource Center for Research (KXRCR000001) manual. All the incubation solutions were changed daily. Aliquots of 40 nL mRNA solutions were prepared, and the mRNAs were pulled with glass capillary tubing (1.5–2.0 mm in diameter) by using a 10-nL nanoinjector (VWR Scientific, USA). Then, 50 ng/50 nl of hPKD2L1 mRNA was injected per one oocyte to make hPK2L1-injected oocytes. After that, 50 ng/50 nl of hPKD2L1 R299A mRNA was injected per one oocyte to make hPK2L1-R299A-injected oocytes. Finally, 50 nl of mRNA-free PBS was injected per one oocyte to make non-injected oocytes. The electrophysiological recording was performed within 2–7 days of the oocyte isolation.

### Human PKD2L1 Receptor Mutation and In Vitro Transcription of cDNAs

MAX QuikChange Site-Directed Mutagenesis kits (Stratagene, USA) were used for mutating PKD2L1 subunits prior to amplification by PCR. The success of PCR was evaluated using DNA sequencing analysis by Cosmo Gentech Inc. (Korea) after the PCR product was transfected into XL1-Blue super-competent cells, followed by screening. The identified DNA was linearized using the restriction enzyme PmeI, and then transcribed into RNA using T7 In Vitro Transcription kits (Ambion, USA). The final RNA products were resuspended in diethyl pyrocarbonate (DEPC) and stored as aliquots at a final concentration of 1000 ng/μl at –80°C for the next experiment.

### Molecular Docking Study with Three-Dimensional (3D) Modeling

For the molecular docking study of the PKD2L1 and LPGA interaction, the protein structure was obtained from the (PDB) https://www.rcsb.org/; the PDB ID of the selected protein structure was 5Z1W. The 3D structure of LPGA was referenced in PubChem (CID code: 57339449). The docking study was performed in a basic setting using AutoDock Tools (Scripps Research Institute [version 4.2.6], USA). The performance state of the protein was enhanced by removing water molecules from the macromolecule, adding polarity and hydrogen ions, and computing the Gasteiger charges. The models were selected on the basis of intermolecular energy, inhibition constant, binding structures, and binding energy. The complex of LPGA and PKD2L1 was analyzed using LIGPLOT, which calculated the binding activity between LPGA and PKD2L1. The distance between the PKD2L1 and LPGA molecule interaction site was measured using PyMol.

### Data Recording

An oocyte clamp (OC-725C; Warner Instruments, USA) with a perfusion chamber was used for the two-electrode voltage-clamp (TEVC) recordings at room temperature. A recording solution (ND96 bath solution) was prepared as described [27] and applied with LPGA and glutamate during recording, according to the experiment design. Oocytes were placed into the chamber with the ND96 bath solution, flowing at a rate of 2 ml/min. Two electrodes filled with 3M KCl (electrolyte solution, 0.2–0.7 MW resistance) were stabbed at a random position in every oocyte. Experiments were set with a –70 mV holding potential for the current recording and –100 to+60 mV within 300 ms for ramping the voltage relationship of PKD2L1s. All the data were collected and analyzed using Digidata 1320 (Molecular Devices, USA) and pCLAMP 9 software (Axon Instruments, USA).

### Data Analysis

Voltage-clamp recording was performed to investigate how the LPGA-induced inward current (*I_LPGA_*) behaves according to the concentration of LPGA. The results of these data recordings were analyzed using Origin19b (Origin, USA). The relationship between the concentration of LPGA was fit by the Hill equation: y = Vmax × [x]^n^/([EC_50_]^n^ + [x]^n^), where y is the peak current at a given concentration of LPGA, Vmax is the maximal peak value, EC_50_ is the half-maximal activation concentration of LPGA on *I_LPGA_*, [x] is the concentration of LPGA, and n is the interaction coefficient. All the values are presented as the standard error of the mean. The differences between the means of both values were determined using unpaired Student’s *t*-tests. *p* <0.05 was considered statistically significant.

## Results

### Confirmation of LPGA Induced Inward-Current against Human PKD2L1

PKD2L1 is a known receptor for detecting sour taste among the five flavors that mammals, including humans, can distinguish. To find major molecules in metabolites that can affect taste and produced as kimchi ferments, a screening assay was conducted. We found that the LPGA in metabolites showed a significant regulation on the activity of hPKD2L1. Therefore, physiological activity and molecular biological binding were confirmed through TEVC experiment and molecular biological experiment method. This study shows that LPGA induced inward-current activity and its activity as a ligand in hPKD2L1 was studied. Fig. 1A shows the chemical structure of LPGA, which is a free amino group of glutamic acid or glutamine that is cyclized to form lactam [28]. Activity against hPKD2L1 can be confirmed by proton [29]. As a result of applying different proton concentrations with the ND96 bath solution, it was confirmed that hPKD2L1 showed activity dependent on proton concentration. As shown in Fig. 1C, and confirming that the activity by the above proton is specific to hPKD2L1, no activity was observed when protons were applied on the non-injected group. The results of using LPGA in the no-injection group also revealed no activity. In addition, Fig. 1D shows that hPKD2L1 is activated by protons and LPGA. Therefore, LPGA was confirmed as a ligand for hPKD2L1.

### Confirmation Induced-Inward Current by LPGA Has a Concentration-Dependent and Voltage-Independent Manner to Human PKD2L1

**Fig. 2:**
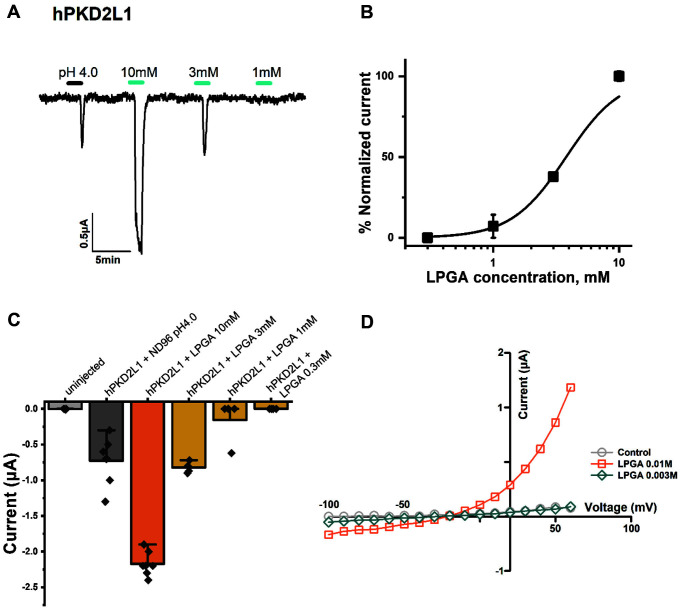
Confirmation of LPGA's activation manner on hPKD2L1. (**A**) Confirm whether this activity has concentration-dependent by applying various concentrations of LPGA to hPKD2L1. (**B**) The sigmoidal graph is shown by normalizing the current shown in 2A, the current of 10 mM LPGA was specified as 100%, and pitted using the Hill equation. (**C**) The current activity of LPGA applied to hPKD2L1 is shown in a vertical bar chart. Each 'n' number is represented by a black rhombus shape and the applied concentration was indicated at the top of the bar. (**D**) A ramp protocol was performed to confirm the voltage-dependent manner, and the voltage was applied from -100 mV to +60 mV. Each applied concentration is shown in the figure. All experiments were performed at room temperature, and the holding potential of the protocol used in the experiments was -80 mV. (*n* = 6-8, obtained from four different oocytes)

hPKD2L1 was confirmed to be a receptor activated by protons and a specific chemical called LPGA. To verify this activating mechanism, LPGA was applied at different concentrations to hPKD2L1. In Fig. 2A, to confirm the hPKD2L1 expression in oocytes, ND96 (pH 4.0) was applied using different concentrations of 10 mM, 3 mM, and 1 mM of LPGA. The result confirmed that this activity has a concentration-dependent manner. Fig. 2B shows the results of normalizing that concentration-dependent activity in Fig. 2A. In Fig. 1, LPGA and protons did not induce an inward current in non-injected oocytes, but did so in hPKD2L1- injected oocytes. This result shows that hPKD2L1 has a specific binding site with LGPA in a concentration-dependent manner. Fig. 2C shows the result of the LPGA-inducing current expressed in numerical data and presented in a vertical error-bar chart. LPGA 10 mM induced about 2.98 folds more activity than ND96 (pH 4.0), and 3mM induced about 1.12 folds more activity. Fig. 2D shows whether the activity of hPKD2L1 by LPGA was voltage-dependent, with fluctuating voltages from -100 mV to +60 mV applied. As a result, it was confirmed that it is a voltage-independent manner in which the slope does not change due to voltage fluctuations and that the current is affected by the ligand, LPGA. The reverse potential was between -20 mV and -10 mV.

### 3D Docking Modeling of Human PKD2L1 Receptor Interacting with LPGA

**Fig. 3:**
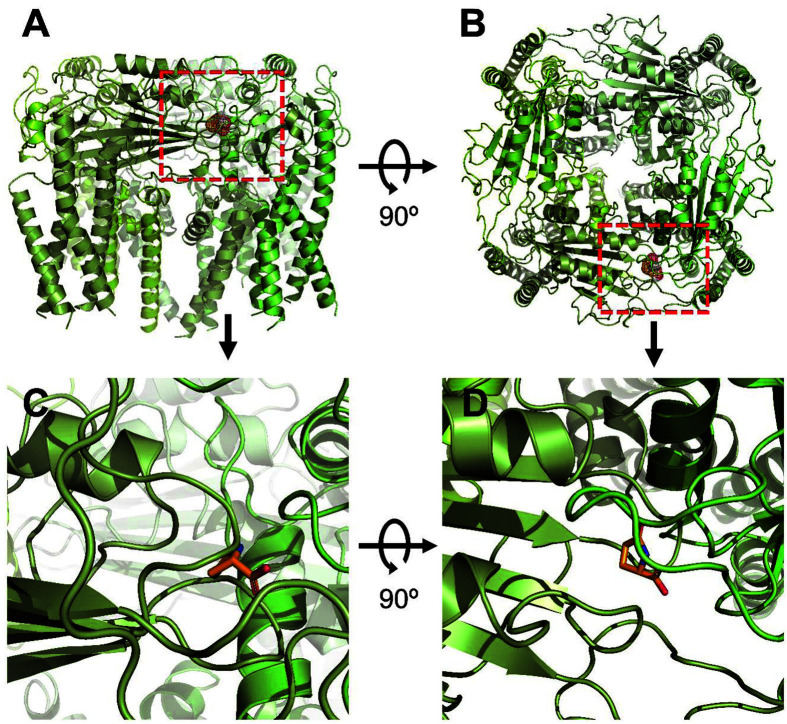
Molecular docking modeling of LPGAs to hPKD2L1. (**A**) Side views of the docked LPGAs in complex with hPKD2L1. (**B**) Top views of the docking model. (**C**) An enlarged view of the red dotted line in Fig. 3A. (**D**) An enlarged view of the red dotted line in Fig. 3B.

**Fig. 4:**
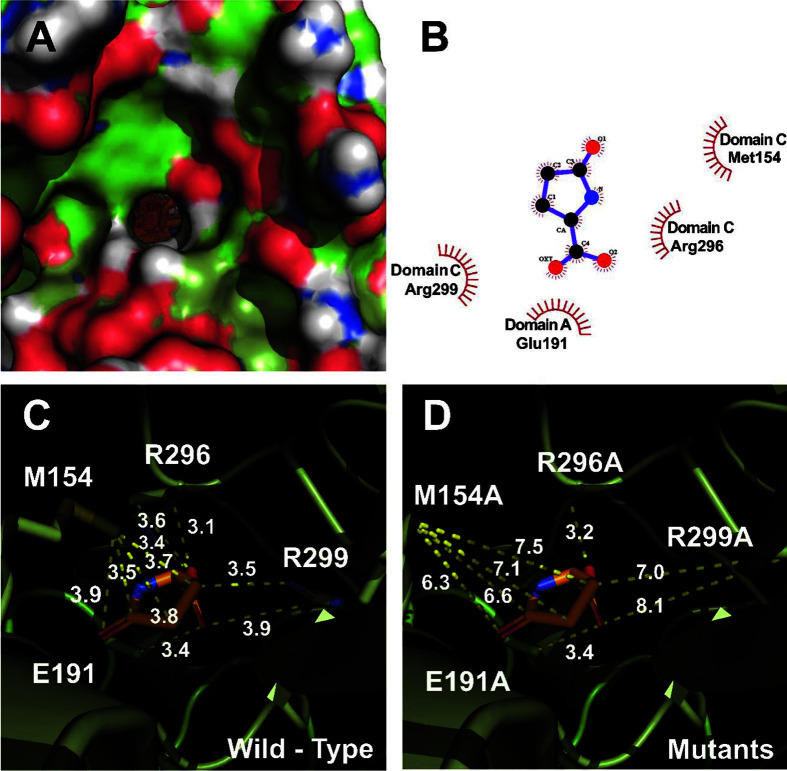
The binding pocket and docking results of LPGAs and hPKD2L1. (**A**) The binding pocket in the hPKD2L1 region of the extracellular domain membrane pocket side. (**B**) Two-dimensional schematic presentation of the predicted binding mode of LPGAs in the ligand-binding pocket. The ligands and important residues are shown. (**C, D**) Computational simulated binding interaction of ligand and residues in wild-type and mutants. The replaced mutants showed changes in interaction activities at varying degrees. (**C**) Interaction between naringin and wild-type hPKD2L1. (**D**) Interaction between LPGAs and mutant-type hPKD2L1.

Fig. 3 shows the LPGA binding site on the human PKD2L1 receptor through 3D protein modeling. Unlike ortholog PKD2, hPKD2L1 adopts an open conformation, in which the stomatal helix (PH) and transmembrane segment 6 (S6) of PKD2L1 are involved in upper and lower gate opening. Structural comparison of PKD2L1 and PKD2-based homology models suggests that pore domain expansion is coupled with structural changes in the voltage sensing domain (VSD) through a series of π–π interactions as a gating mechanism [30]. In Fig. 4, in-silico protein-ligand complex modeling was performed to confirm the interaction site of LGPA on the human PKD2L1 receptor. The interaction with LPGA was implemented as a 3D model based on the crystal structure information of human PKD2L1 (PDB ID: 5Z1W) and indicates the position of a residue to consider as a binding pocket in the most stable energy state. The interaction distances of LPGA with each residue of wild-type and mutant hPKD2L1 receptors are shown in Figs. 4C and 4D. The binding energy was –6.47 kcal/mol; the intermolecular energy was –7.07 kcal/mol; the internal energy was –0.19 kcal/mol; the torsional energy was 0.6 kcal/mol; the unbound extended energy was –0.19 kcal/mol; refRMS was 203.65; and Ki was 18.14 μM. The interatomic distances of R299 changed from 3.5, and 3.9 Å to 7.0, and 8.1 Å of mutant R299A.

### Confirmation of Binding Site of LPGA by Mutant-Type hPKD2L1

**Fig. 5:**
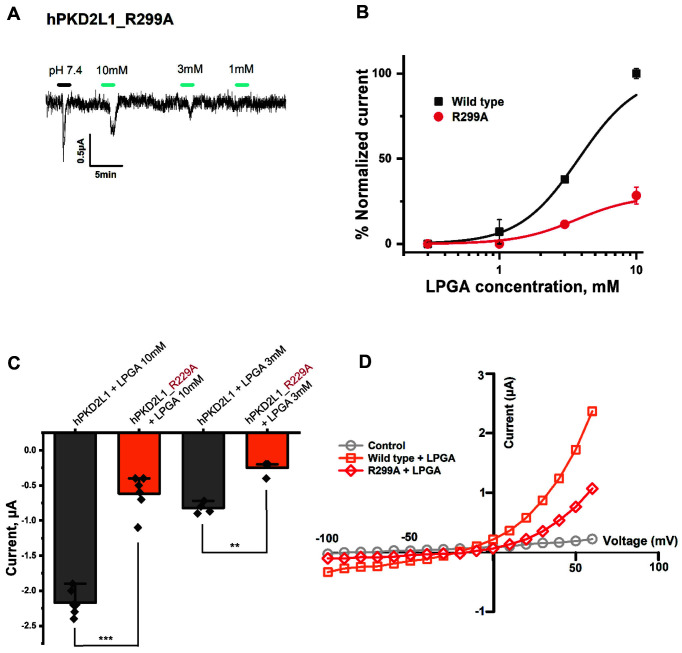
Confirmation of inward current change through a changing promising binding residue of LPGA in hPKD2L1 mutant-type. (**A**) Result of applying ND96 (pH4.0) and LPGA to R229A mutant-type by concentration. The scaling bar and each applied pH are displayed at the top side of the current in the figures. (**B**) Results of normalization of inward current by Fig. 5A that LPGA and comparison with wild-type inward current. This sigmoid graph was pitted by the Hill equation. (**C**) The inward current of wild-type and mutant-type hPKD2L1 was numerically quantified and expressed as a vertical error-bar chart. Each 'n' number is represented by a black rhombus shape. The *p*-value is < 0.001 *** and < 0. 0.01 **. (**D**) Current of wild-type and mutant-type according to voltage fluctuation from -100 mV to +60 mV through ramp protocol. All experiments were performed at room temperature, and the holding potential of the protocol used in the experiments was - 80 mV. (*n* = 6-8, obtained from four different oocytes)

Through the results up to Fig. 4, the interaction between hPKD2L1 and LPGA and its activation mechanism was confirmed. To confirm the binding site of LPGA in hPKD2L1, a wild-type crystal structure was obtained in silico, and several candidate binding sites of LPGA were obtained with the most stable energy through protein-ligand docking modeling. Among these residues, the point mutation experiment was performed to confirm the change in the binding activity of LPGA when the amino acid of the most potent binding site was changed. In Fig. 5A, LGPA's candidate binding site in hPKD2L1 was mutated, injected, and expressed in oocytes, and LPGA-induced inward-current changes were confirmed by TEVC. As a result, when the R299 residue was changed to alanine, a different inward current from the wild-type was confirmed. R229A mutant-type had a current by LPGA that was reduced more than the wild-type. Since this reduced current showed the greatest decrease compared to other promising mutant types, it was confirmed that the R299 residue was the binding site for LPGA. In Fig. 5B, current normalization was performed to compare the wild-type and mutant-type by applying LPGA. In Fig. 5C, the current activity of the wild-type and mutant-type LGPA of hPKD2L1 was compared with numerical data. Wild-type 10 mM LPGA showed activity of about -2.17 μA, and mutant-type about -0.61 μA, while wild-type 3 mM LPGA showed about -0.82 μA, and mutant-type about -0.25 μA. As a result, in the mutant-type LPGA the activity of hPKD2L1 was reduced by about 3.52 folds (28% current compared to wild-type) by 10mM LPGA, and reduced 3.29 folds (30% current compared to wild-type) by 3mM LPGA. In Fig. 5D, using the ramp protocol, the effect of the fluctuation voltage of wild-type and mutant-type was confirmed. As a result, it was confirmed that the mutant-type had a reduced current compared to the wild-type.

## Discussion

Our goal in the present work was to analyze the interaction mechanism between L-pyroglutamic acid (LPGA) and human sour taste receptors at the cellular molecular level. To this end, hPKD2L1, discovered by Dr. Charles Zuker [31], was injected into *Xenopus* oocytes as mRNA and expressed. We succeeded in expressing the sour taste receptor in *Xenopus* oocytes and performed whole-cell recording of hPKD2L1-expressing oocytes with two-electrode voltage clamps. LPGA was found to be an agonist activating hPKD2L1. To find the binding site, candidates with low binding energy were derived through computer simulation, and mutants were created by point-mutating the candidates to alanine. Similarly, among the binding site candidates mutated by whole-cell recording, a binding site that actually reduced the binding force of LPGA was found.

This study demonstrated for the first time that LPGA, one of the active ingredients of kimchi, binds to hPKD2L1 expressed on the human tongue to generate a sour taste signal. Of course, there is a limitation that the data are not confirmed from actual human taste cells. This paper has no data showing that LPGA has a sour taste through sensory evaluation. Here, through our experiments, it was confirmed that LPGA binds to hPKD2L1 at the cellular molecular level to generate signals in taste cells. Taking these facts together, it can be seen that when LPGA is ingested, a signal generated by combining with hPKD2L1, a membrane protein present in human taste cells, is transmitted to the brain and recognized as a sour taste.

One of the hardest things in the food industry is keeping the taste the same. This is because taste has a strong subjective tendency and there was no way to objectively quantify it. Attempts have been made to quantify taste. Among them, an electronic tongue and an electronic nose that can be quantified through various sensors using a chemical approach have been developed [32, 33]. As a method for measuring the taste of the electronic nose and tongue, there is a method of measuring the change in current caused by the redox reaction occurring at the electrode through an electrochemical sensor and a method of using biomaterials, such as enzyme reaction or antibody-antigen reaction. Although it has the advantage of easy measurement and high sensitivity, it was clear that the distortion of the result or the limitation of the durability of the sensor and the analyte were limited. So, in this field, we are challenging research and development to develop an electronic tongue and an electronic nose through taste receptors.

With the method used in the current study, substances that bind to taste receptors can be screened and the activation mechanism analyzed. Using this research method, qualitative quantification is possible. The results of the current study alone have limitations. Since the result was confirmed by expression in *Xenopus* oocytes instead, it cannot be considered the same as the quantitative value felt by an actual person. However, if the data are accumulated, it is possible to convert the comparison to be similar to the quantitative value in the human standard through calculation. Through this study, it is also possible to see the intensity of taste as an objective value and to know the change in intensity of taste according to the concentration.

In conclusion, through the results of the present paper, it was confirmed that R299 of hPKD2L1 is a position involved in the binding of LPGA. We show that LPGA interacts with hPKD2L1 in a concentration-dependent and voltage-independent manner. These results mean that LPGA, one of the active ingredients of kimchi, has a sour taste. The findings help us understand how sour taste receptors work and suggest the possibility of modulating them. Above all, this study suggests the possibility of objectifying the intensity of taste.

## References

[1] Breslin PA, Spector AC (2008). Mammalian taste perception. Curr. Biol..

[2] Drewnowski A, Mennella JA, Johnson SL, Bellisle F (2012). Sweetness and food preference. J. Nutr..

[3] Glendinning JI (1994). Is the bitter rejection response always adaptive?. Physiol. Behavior..

[4] Huang AL, Chen X, Hoon MA, Chandrashekar J, Guo W, Tränkner D (2006). The cells and logic for mammalian sour taste detection. Nature.

[5] Adler E, Hoon MA, Mueller KL, Chandrashekar J, Ryba NJ, Zuker CS (2000). A novel family of mammalian taste receptors. Cell.

[6] Chandrashekar J, Hoon MA, Ryba NJ, Zuker CS (2006). The receptors and cells for mammalian taste. Nature.

[7] Zhang Y, Hoon MA, Chandrashekar J, Mueller KL, Cook B, Wu D (2003). Coding of sweet, bitter, and umami tastes: different receptor cells sharing similar signaling pathways. Cell.

[8] Nelson G, Hoon MA, Chandrashekar J, Zhang Y, Ryba NJ, Zuker CS (2001). Mammalian sweet taste receptors. Cell.

[9] Zhao GQ, Zhang Y, Hoon MA, Chandrashekar J, Erlenbach I, Ryba NJ (2003). The receptors for mammalian sweet and umami taste. Cell.

[10] Chandrashekar J, Mueller KL, Hoon MA, Adler E, Feng L, Guo W (2000). T2Rs function as bitter taste receptors. Cell.

[11] Mueller KL, Hoon MA, Erlenbach I, Chandrashekar J, Zuker CS, Ryba NJ (2005). The receptors and coding logic for bitter taste. Nature.

[12] Chandrashekar J, Kuhn C, Oka Y, Yarmolinsky DA, Hummler E, Ryba NJ (2010). The cells and peripheral representation of sodium taste in mice. Nature.

[13] Park K-Y, Jeong J-K, Lee Y-E, Daily III JW (2014). Health benefits of kimchi (Korean fermented vegetables) as a probiotic food. J. Med. Food.

[14] Park JM, Shin JH, Gu JG, Yoon SJ, Song JC, Jeon WM (2011). Effect of antioxidant activity in kimchi during a short-term and over-ripening fermentation period. J. Biosci. Bioeng..

[15] Woo M, Kim MJ, Song YO (2018). Bioactive compounds in kimchi improve the cognitive and memory functions impaired by amyloid beta. Nutrients.

[16] Kim B, Song JL, Ju JH, Kang SA, Park KY (2015). Anticancer effects of kimchi fermented for different times and with added ingredients in human HT-29 colon cancer cells. Food Sci. Biotechnol..

[17] Kwak S-H, Cho Y-M, Noh G-M, Om A-S (2014). Cancer preventive potential of kimchi lactic acid bacteria (*Weissella cibaria*, *Lactobacillus plantarum*). J. Cancer Prev..

[18] Park K-Y, Baek K-A, Rhee S-H, Cheigh H-S (1995). Antimutagenic effect of kimchi. Food Sci. Biotechnol..

[19] Pfeiffer P, König H (2009). Pyroglutamic acid: a novel compound in wines. Biology of Microorganisms on Grapes, in Must and in Wine.

[20] Aiello A, Pepe E, De Luca L, Pizzolongo F, Romano R (2022). Preliminary study on kinetics of pyroglutamic acid formation in fermented milk. Int. Dairy J..

[21] Mucchetti G, Locci F, Massara P, Vitale R, Neviani E (2002). Production of pyroglutamic acid by thermophilic lactic acid bacteria in hard-cooked mini-cheeses. J. Dairy Sci..

[22] Jiménez-Arias D, Garcia-Machado FJ, Morales-Sierra S, Luis JC, Suarez E, Hernández M (2019). Lettuce plants treated with Lpyroglutamic acid increase yield under water deficit stress. Environ. Exp. Bot..

[23] Wang M, Chen J, Lin X, Huang L, Li H, Wen C (2021). High humidity aggravates the severity of arthritis in collagen-induced arthritis mice by upregulating xylitol and *L*-pyroglutamic acid. Arthritis Res. Ther..

[24] Gazme B, Boachie RT, Tsopmo A, Udenigwe CC (2019). Occurrence, properties and biological significance of pyroglutamyl peptides derived from different food sources. Food Sci. Human Wellness.

[25] Diepeveen J, Moerdijk‐Poortvliet TC, van der Leij FR (2022). Molecular insights into human taste perception and umami tastants: a review. J. Food Sci..

[26] Zhang Y, Venkitasamy C, Pan Z, Liu W, Zhao L (2017). Novel umami ingredients: Umami peptides and their taste. J. Food Sci..

[27] Guan B, Chen X, Zhang H (2013). Two-electrode voltage clamp. Ion Channels.

[28] Kumar A, Bachhawat AK (2012). Pyroglutamic acid: throwing light on a lightly studied metabolite. Curr. Sci..

[29] Chang RB, Waters H, Liman ER (2010). A proton current drives action potentials in genetically identified sour taste cells. Proc. Natl. Acad. Sci. USA.

[30] Su Q, Hu F, Liu Y, Ge X, Mei C, Yu S (2018). Cryo-EM structure of the polycystic kidney disease-like channel PKD2L1. Nat. Commun..

[31] Chandrashekar J, Yarmolinsky D, von Buchholtz L, Oka Y, Sly W, Ryba NJ (2009). The taste of carbonation. Science.

[32] Chen Y, Wei Z, Zhang T, Ng KH, Ye J, He W (2022). Physicochemical, electronic nose and tongue, sensory evaluation determination combined with chemometrics to characterize Ficus hirta Vahl. (Moraceae) beer. J. Food Qual..

[33] Deisingh AK, Stone DC, Thompson M (2004). Applications of electronic noses and tongues in food analysis. Int. J. Food Sci. Technol..

